# Structural Basis of Transcriptional Gene Silencing Mediated by *Arabidopsis* MOM1

**DOI:** 10.1371/journal.pgen.1002484

**Published:** 2012-02-09

**Authors:** Taisuke Nishimura, Guillaume Molinard, Tom J. Petty, Larissa Broger, Caroline Gabus, Thanos D. Halazonetis, Stéphane Thore, Jerzy Paszkowski

**Affiliations:** 1 Department of Plant Biology, University of Geneva, Geneva, Switzerland; 2 Department of Molecular Biology, University of Geneva, Geneva, Switzerland; University of Michigan, United States of America

## Abstract

Shifts between epigenetic states of transcriptional activity are typically correlated with changes in epigenetic marks. However, exceptions to this rule suggest the existence of additional, as yet uncharacterized, layers of epigenetic regulation. MOM1, a protein of 2,001 amino acids that acts as a transcriptional silencer, represents such an exception. Here we define the 82 amino acid domain called CMM2 (Conserved MOM1 Motif 2) as a minimal MOM1 fragment capable of transcriptional regulation. As determined by X-ray crystallography, this motif folds into an unusual hendecad-based coiled-coil. Structure-based mutagenesis followed by transgenic complementation tests in plants demonstrate that CMM2 and its dimerization are effective for transcriptional suppression at chromosomal loci co-regulated by MOM1 and the siRNA pathway but not at loci controlled by MOM1 in an siRNA–independent fashion. These results reveal a surprising separation of epigenetic activities that enable the single, large MOM1 protein to coordinate cooperating mechanisms of epigenetic regulation.

## Author Summary

Epigenetic shifts in transcriptional activities are usually correlated with changes in chromatin properties and covalent modification of DNA and/or histones. There are, however, exceptional regulators that are able to switch epigenetic states without the apparent involvement of changes in chromatin or DNA modifications. MOM1 protein, derived from CHD3 chromatin remodelers, belongs to this group. Here we defined a very small domain of MOM1 (less than 5% of its total sequence) that is sufficient for epigenetic regulation. We solved the structure of this domain and found that it forms a dimer with each monomer consisting of unusual consecutive 11 amino-acid hendecad repeats folding into an antiparallel coiled-coil. *In vivo* experiments demonstrated that the formation of this coiled-coil is essential for silencing activity; however, it is effective only at loci co-silenced by MOM1 and small RNAs. At loci not controlled by small RNAs, the entire MOM1 protein is required. Our results demonstrate that a single epigenetic regulator is able to differentially use its domains to control diverse chromosomal targets. The acquisition of the coiled-coil domain of MOM1 reflects a neofunctionalization of CHD3 proteins, which allowed MOM1 to broaden its activity and to provide input into multiple epigenetic pathways.

## Introduction

Transcriptional gene silencing (TGS) refers to the stable repression of transcription and mainly affects transposons, chromosomal repeats and transgenic inserts; however, it may also suppress the expression of certain protein-coding genes. In multicellular eukaryotes, TGS can persist through mitotic divisions and also be inherited meiotically, which is especially well documented for plants. Such TGS stability is achieved by the concerted action of multiple epigenetic mechanisms that establish and maintain particular patterns of covalent modification of DNA and histone proteins throughout DNA replication [1]. For example, local hypermethylation of cytosines accompanied by repressive marks on histones, such as di-methylation of histone H3 at lysine in position 9 (H3K9me2), shifts chromatin structure into a repressive conformation and results in TGS. Conversely, decreased levels or loss of cytosine methylation (^m^C) and H3K9me2 release silencing [1].

In plants, ^m^C occurs in two classes of sequence context; CG and non-CG. CG methylation (^m^CG) is propagated by the DNA methyltransferase MET1 in cooperation with SRA domain proteins, which use newly replicated, hemi-methylated DNA as template [2]–[5]. Non-CG methylation can be further subdivided into ^m^CHG and ^m^CHH (where H stands for C, A or T) maintained by two additional DNA methyltransferases: CMT3 and DRM2, respectively. CMT3 uses the H3K9me2 mark for methylation targeting [6]–[8], while DRM2 is recruited to its targets by siRNAs in an RNA-directed DNA methylation (RdDM) process [9]. ^m^CHG and H3K9me2 are interlinked by a complex regulatory loop [10] in which the H3K9 histone methyltransferase SUVH4/KYP uses CHG methylation for targeting and creates H3K9me2-rich domains attracting CMT3 [11]–[13]. Moreover, global reduction of cytosine methylation in *met1* mutants causes redistribution of H3K9me2 and release of TGS [14]–[16].

*Arabidopsis* MORPHEUS' MOLECULE1 (MOM1) is an exceptional TGS regulator that acts largely independently of changes in the levels of DNA methylation and H3K9me2. In *mom1* mutants, although TGS is released at transposons, repetitive sequences and transgenes, silencing release occurs in this case without major alterations in DNA or histone modification, as is the case for the mutation of genes necessary for maintenance of cytosine and histone methylation [17]–[21]. Therefore, MOM1 appears to control TGS using different, as yet not well-understood molecular mechanisms. The results from recent studies on genetic modifiers of the *mom1* mutation suggest that MOM1 acts downstream of RdDM-mediated cytosine methylation [22], [23]. However, this occurs only at a subset of loci subjected to MOM1-mediated regulation; TGS activity at other loci was either independent of RdDM, or MOM1 was able to modify the activity of RdDM (enhancing or suppressing) [23]. Thus, these results reveal complex cooperation between MOM1 and the RdDM in the regulation of TGS, dividing their common target loci into several categories according to their independence or cooperation with MOM1 and RdDM-mediated regulation [23]. In addition, the preferential targets for MOM1-mediated TGS are loci associated with intermediate levels of both H3K9me2 and H3K4me2, which marks transcriptionally active chromatin [20]. Interestingly, at one particular locus targeted by RdDM, *SUPPRESSOR OF drm1 drm2 cmt3* (*SDC*), H3K9me2 levels decreased in *mom1* mutants, suggesting that MOM1 is involved in the transduction of RdDM signals to H3K9me2 marks at the *SDC* gene [22]. The results from these previous studies all point towards several distinct mechanisms of MOM1-mediated TGS that appear to be executed according to epigenetic marks on the target loci, in cooperation with further epigenetic regulatory mechanisms.

MOM1 is a large nuclear protein of 2001 amino acids containing an incomplete and highly degenerate helicase domain related to a similar domain found in CHD3 chromatin-remodeling factors [17], [24]. However, functional studies of MOM1 deletions showed this fraction of the protein to be dispensable for its TGS activity [24]. Surprisingly, only the predicted nuclear localization signal (NLS) and a short fragment of MOM1 of less than 200 amino acids (1663 to 1859) containing a conserved plant-specific motif of 82 amino acids (1734 to 1815), named Conserved MOM1 Motif 2 (CMM2), is required for TGS activity [24]. CMM2 is found in MOM1 homologues of all vascular plants for which genome sequences are available. This implies that the CMM2 domain plays a crucial role in TGS regulation in most land plants; however, the molecular mechanism of CMM2-mediated silencing remains obscure.

Here we present the crystal structure of the CMM2 domain and the results of *in vivo* studies that indicate the importance of CMM2 homo-multimerization for its TGS activity. The structural analyses uncover intermolecular interactions between CMM2 domains via the formation of an anti-parallel coiled-coil structure and suggest the formation of multimers. Testing these predictions *in vivo*, we have confirmed that CMM2 interactions do occur and are essential for the CMM2-mediated activity in TGS. Moreover, we found that, although CMM2 was able to mediate TGS at loci regulated by MOM1 in cooperation with RdDM, CMM2 silencing activity was compromised at loci controlled by MOM1 in an RdDM-independent fashion. The analysis of MOM1 mutants with a disrupted CMM2 intermolecular interface in transgenic plants revealed that CMM2 intermolecular interactions are necessary and largely sufficient for TGS regulation at loci that are also regulated by RdDM. However, for the initiation and/or maintenance of TGS at loci not controlled by RdDM, the coiled-coil forming CMM2 domain is only partially effective. Thus, further elements of MOM1, or possibly the entire protein, are required here for stabilization of TGS. Thus, our results provide a molecular framework for understanding how MOM1 mediates TGS.

## Results

### The CMM2 domain is necessary and sufficient for silencing of a transgenic locus

**Figure 1:**
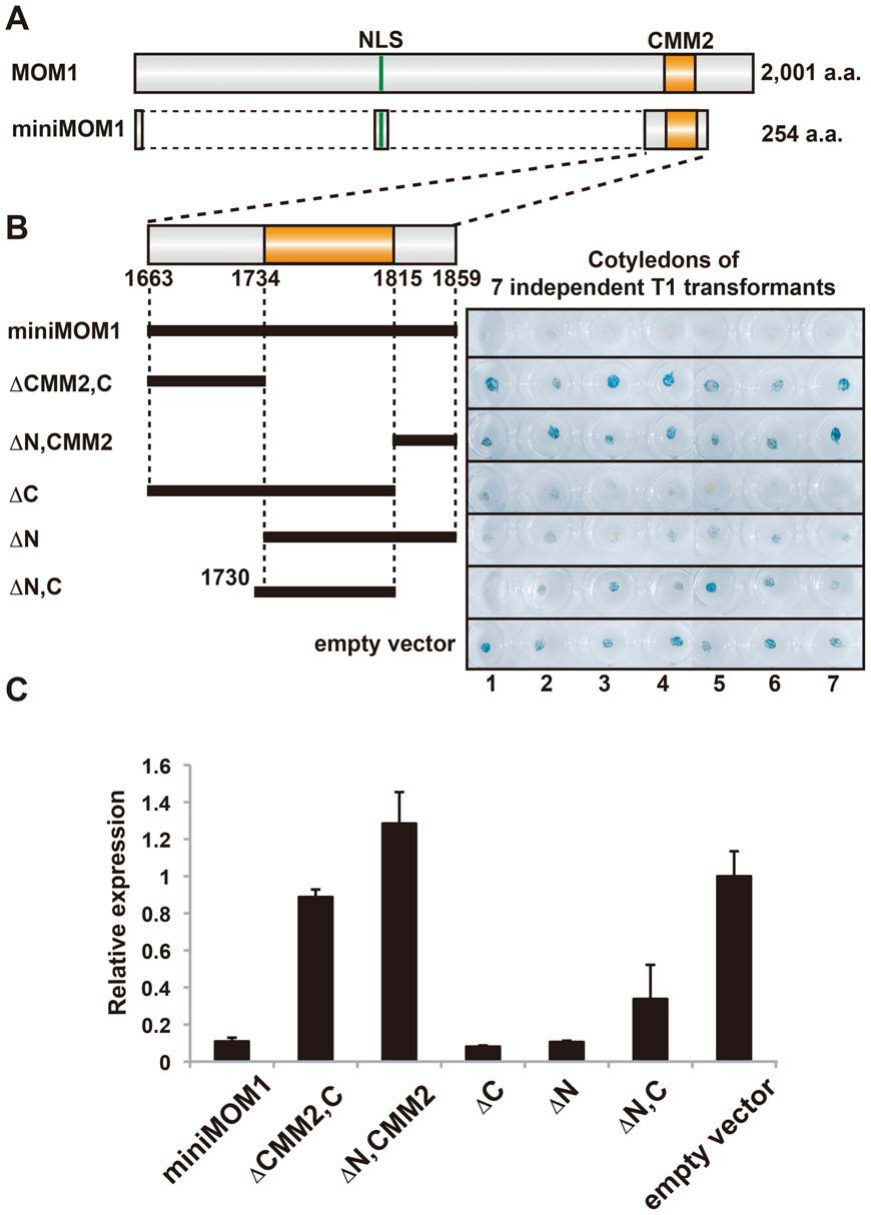
CMM2 is necessary and sufficient for the TGS activity of MOM1. (A) Schematic representation for MOM1 and miniMOM1. (B) *Left*, deletion derivatives of miniMOM1 that were introduced to *mom1* L5 plants harboring a transgenic L5 locus encoding ß-glucuronidase [25]. *Right*, histochemical GUS staining of cotyledons of seven independent 1-week-old T1 transgenic plants transformed with the corresponding miniMOM1 deletion derivatives. The “empty vector” control corresponds to *mom1* L5 transformed with a vector construct without miniMOM1 sequences. (C) Relative levels of *GUS* mRNA in T2 plants from 3 independent T1 plants determined by quantitative RT-PCR and normalized to 18S rRNA. The mean of the “empty vector” control was set to 1. Error bars represent S.E. calculated from 3 experimental sets of 40 to 50 plants each.

Previously reported functional mapping of the MOM1 protein demonstrated that a short fragment of the protein containing CMM2, known as miniMOM1 (Figure 1A), was necessary and sufficient for TGS [24]. Although these results strongly suggested that CMM2 is the crucial domain for the TGS activity of MOM1, a contribution to the silencing activity of miniMOM1 regions adjacent to CMM2 (N- terminal amino acids 1663 to 1733, and C- terminal amino acids 1816 to 1859) (Figure 1B) could not be ruled out [24]. To address more precisely the TGS activity of the CMM2 domain only, we removed fragments of miniMOM1 flanking CMM2 and performed transgenic complementation assays for restoration of TGS in *mom1* mutant plants (Figure 1B and 1C). As readout, we used the well-characterized transgenic locus coding for ß-glucuronidase (GUS), which is known to be silenced by TGS (L5 line [25]). Transcription of this locus was restored in *mom1* mutants and the GUS activity visualized by histochemical staining (*mom1*L5 line [17], [24]). In the case of functional complementation of the *mom1* mutation, the *GUS* locus was re-silenced and its transcript and GUS activity vanished.

To monitor GUS activity, we stained cotyledons of seven T1 transgenic plants obtained with each complementation construct (Figure 1B). The original miniMOM1 construct suppressed GUS activity, confirming the previous observations (Figure 1B, “miniMOM1”; [24]). The *GUS* gene remained active in transgenic plants with chromosomal integrations of the empty expression cassette (Figure 1B, “empty vector”). Incorporation of miniMOM1 fragments flanking CMM2 into the expression cassette had no influence on GUS activity, suggesting that these parts of miniMOM1 have no TGS activity of their own (Figure 1B, ΔCMM2, C” and “ΔN,CMM2” constructs). Consistently, deletions of these regions from miniMOM1 (Figure 1B, “ΔC” and “ΔN” constructs) did not affect TGS efficiency. Finally, we assessed the TGS activity of the CMM2 domain itself (Figure 1B “ΔN,C” construct) and found a somewhat variable degree of *mom1* complementation, with three transgenic plants displaying a high degree of complementation (Figure 1B, plants 1, 2, 7), three with incomplete complementation (Figure 1B, plants 3, 4, 6) and one with no indication of complementation (Figure 1B, plant 5).

To better quantify the TGS activity of miniMOM1 derivatives and especially of the CMM2 domain alone, we performed quantitative RT-PCR analyses. For each construct, we examined the T2 progeny of three randomly chosen T1 plants (Figure 1C). “miniMOM1”, “ΔC” and “ΔN” complemented the *mom1* mutation and suppressed *GUS* transcription (Figure 1C), confirming the results from our histochemical analyses. Also consistent with the GUS staining results, deletion of CMM2 abolished TGS activity (Figure 1C, “ΔCMM2, C” and “ΔN, CMM2”). The CMM2 domain alone retained silencing activity, although with slightly lower and a more variable TGS efficiency (Figure 1C, “ΔN, C”), which was mainly due to the differences among independent transgenics in transcript levels for this particular construct (Figure S1). Therefore, we conclude from this set of results that CMM2 is necessary and sufficient for the restoration of TGS at the transgenic *GUS* locus.

### CMM2 domains form an anti-parallel coiled-coil structure

**Figure 2:**
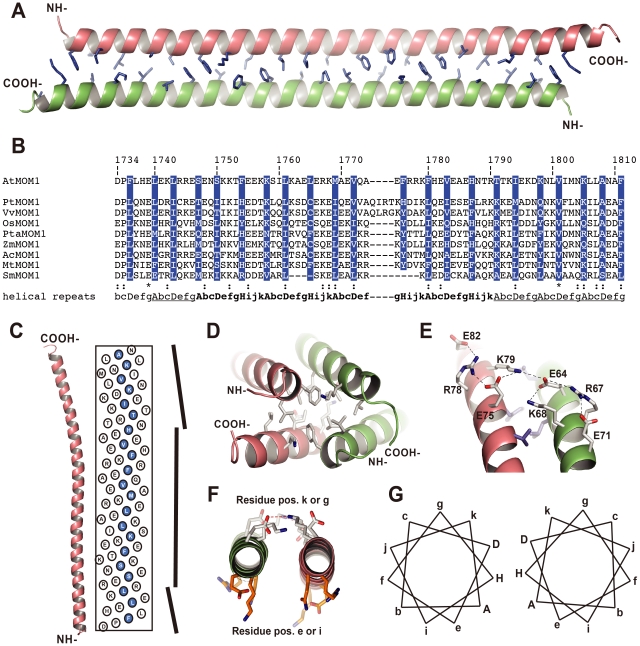
Crystal structure of the CMM2 protein fragment. (A) Overall view of the CMM2 coiled-coil structure. The two monomers are depicted as orange- and green-colored ribbons and the residues forming the interface are shown using the stick representation and colored in blue. (B) Sequence alignment of the different CMM2 sequences found in plants [24]. At - *Arabidopsis thaliana*, Pt - *Populus trichocarpa*, Vv - *Vitis vinifera*, Os - *Oryza sativa*, Pta - *Pinus taeda*, Zm - *Zea mays*, Ac - *Aquilegia coerulea*, Mt - *Medicago truncatula*, Sm - *Selaginella moellendorffii.* The hydrophobic residues forming the interphase are highlighted in blue (shown in A). The repeated pattern of amino acids is indicated in letters referring to their positions. Heptad repeats are underlined and hendecad's repeats are in bold. (C) Axial view of the CMM2 monomer structure together with the radial net showing the position of each amino acid on a flat surface. The vertical bars on the right side represent change in the α-helical axis direction. The N-terminus is at the bottom and the C-terminus on top. Amino acids at position A, D and H are highlighted in blue. (D) Hydrophobic interface formed by the tips of the CMM2 monomers. Symmetry-related CMM2 monomers are depicted as orange- and green-colored ribbons. Amino acids involved in the interaction are show as sticks and colored according to their atom types (carbon: white, nitrogen: blue, oxygen, red). (E) View of the intra- and intermolecular salt bridges stabilizing the CMM2 coiled-coil. Amino acids are labeled and depicted as in panel D. Hydrogen bonds are shown as dashed lines. (F) View along the axis of the CMM2 helices illustrating the difference between the orientation of amino acids in positions e or i and in positions k or g. (G) Schematic representation of the amino acid positions in the CMM2 anti-parallel coiled-coil structure. Positions forming the hydrophobic interface are in capital letters. Images were prepared with the program PyMOL (W.L. Delano, The PyMOL Molecular Graphics System, DeLano Scientific, Palo Alto, CA (2002)).

To gain more insight into the function of CMM2, we carried out structural analyses of this domain. A fragment of the MOM1 protein encompassing the most conserved core of CMM2 was expressed in bacteria, purified to homogeneity and crystallized [26]. The best diffracting crystals were obtained with a MOM1 fragment corresponding to amino acid positions 1700 to 1814 (for experimental details, see [26] and Materials and methods) and a complete dataset was collected to a maximal resolution of 3.2 Å (Table S1). The structure was solved using seleno-methionine-containing proteins and the calculated electron density map was of sufficient quality to trace most parts of the CMM2-containing protein fragment. A poor electron density map, most likely the result of the absence of stable secondary structure, prevented us from building the first 28 as well as the last 3 amino acids of the CMM2-containing fragment. Consequently, we focused our attention on the region comprising residues 1729 to 1811, which corresponds almost exactly to the CMM2 motif (Figure 1B and Figure 2B).

The structure reveals that the CMM2 motif folds as a long α-helix. Two such helices form an antiparallel coiled-coil structure (Figure 2A). The asymmetric unit contains two identical coiled-coils that further interact via their N- and C-termini to form the crystal lattice (Figure 2D). Because of their high degree of structural identity, we describe in more detail only one of the two coiled coil structures (Figure 2A).

The mutual association of two CMM2 monomers occurs mainly through a large interface formed by hydrophobic residues (Figure 2A and 2B). Additional stabilization of the coiled-coil comes from an intermolecular network of salt bridges established between glutamate and lysine residues (Figure 2E). In contrast to the classical coiled-coil structure, CMM2 monomers are not wound around each other and, therefore, do not generate a left-handed super-coil. This is different to the superhelical twist commonly observed when a coiled-coil motif is made of heptad repeats, where hydrophobic residues in positions A and D are tightly packed, mimicking knobs into holes [27]. This observation clearly indicates either that the repeated pattern of hydrophobic and polar amino-acids residues is not the classical heptad repeat or that the heptad repeats are separated by insertions, like stutter or stammer. Such insertions are known to reduce left-hand super-coil in coiled-coil structures [28]. By comparing the sequences of CMM2 domains from different plants, it was possible to identify conserved residues belonging to four consecutive 11 amino-acid repeats known as a hendecad (Figure 2B; [29]). These hendecad repeats are preceded by one and followed by three heptad repeats (Figure 2B). The change in the amino-acid repeat length leads to a change in coiled-coil handedness, as depicted in Figure 2C, and results in an almost perfect parallel arrangement of the two helical CMM2 fragments. As observed for hendecad repeats, amino acids located at positions A, D and H form the hydrophobic interface between the two monomers (Figure 2B and 2G).

In general, coiled-coil structures also form higher-order structures using charged residues located at positions other than A, D and H (Figure 2B). Consequently, it is possible that CMM2 forms higher-order multimeric structures *in vivo*. For example, residues located at positions e and i are solvent exposed and potentially accessible for other interactions (Figure 2F). In the context of the crystal lattice, a large surface of interaction is found between the N- and C- termini of CMM2 domains (Figure 2D). This interface is composed almost exclusively of hydrophobic residues and, therefore, may reflect a functional association (Figure 2D). Subsequently, we mutated several residues thought to be essential for the formation of higher-order CMM2 structures to further assess their functional relevance *in vivo*.

### The CMM2 domain forms homodimers *in vivo*

**Figure 3:**
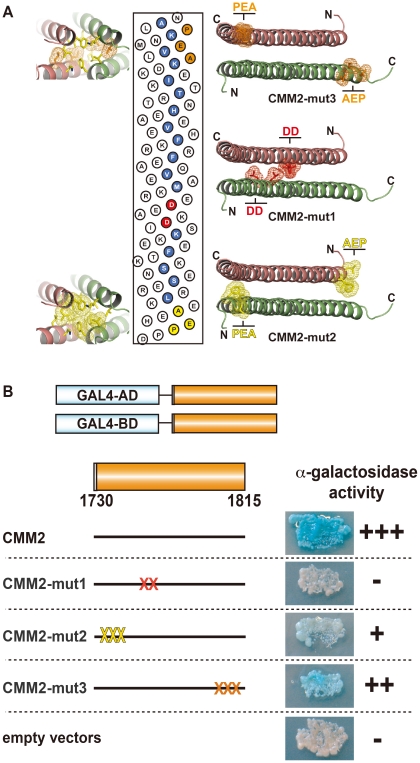
Intermolecular interaction of CMM2 domains *in vivo.* (A) Radial net representation of the CMM2 sequence with the mutated amino acids of the CMM2-mut1, -mut2 and -mut3 constructs indicated in red, yellow and orange, respectively. On each side of the radial net, the CMM2 coiled-coil structure is shown as green- and orange ribbons with the targeted amino acids displayed as sticks surrounded with a mesh surface (colored as described above). (B) Schematic presentation of vectors used in the yeast two-hybrid experiments (*left*) and α-galactosidase staining of yeast co-transformed with corresponding protein fusions (right). “X” on the construct models represents approximate positions of mutations (colored according to A). The “empty vector” contained only GAL4-AD and GAL4-BD.

To examine whether homodimerization of the CMM2 domain observed in the crystal structure also occurs *in vivo*, we performed yeast two-hybrid experiments. We fused the GAL4 activation and the DNA-binding domains to the CMM2 fragment, (aa 1730 to 1815), co-transformed yeast with the constructs encoding both fusion proteins, and monitored their interaction using α-galactosidase staining to assess GAL4-regulated gene expression. While α-galactosidase staining was negative in yeast cells transformed with the empty vectors or with each single GAL4-CMM2 fusion construct, co-transformation of the two constructs strongly activated GAL4 target genes (Figure 3B, construct CMM2). These results are consistent with homodimerization of CMM2 domains *in vivo* and clearly indicate that the determined CMM2 coiled-coil structure is also formed *in vivo*.

Furthermore, based on the crystal structure, the CMM2 monomer not only interacts with itself in a coiled-coil structure, but each CMM2 coiled-coil contributes to the crystal lattice through multiple N- and C-termini interactions (Figure 2D). First, to investigate whether *in vivo* self-interaction of CMM2 is limited to the coiled-coil motif, we introduced point mutations aimed at disrupting the coiled-coil (L1761D and L1765D; CMM2-mut1) (Figure 3A). There was no α-galactosidase activity in yeast cells co-transformed with CMM2-mut1 fusion constructs, indicating the absence of stable interaction between CMM2-mut1 domains (Figure 3B). In contrast, mutations in the N-terminus (F1736P, L1737E, and L1740A; CMM2-mut2) and in the C-terminus (N1799A, I1802E, and L1806P; CMM2-mut3) (Figure 3A), where the coiled-coil dimers interact with each other to form the crystal lattice, led to only slightly lower levels of α-galactosidase activity than with wild-type CMM2 (Figure 3B). In addition, the reduction in α-galactosidase staining was more pronounced for CMM2-mut2 than for CMM2-mut3 (Figure 3B). Mutations in CMM2-mut2 affect two amino acids residing at the end of the coiled-coil structure that form part of the hydrophobic surface of interaction between CMM2 monomers. Therefore, their exchange could alter the coiled-coil structure (Figure 3A), subsequently leading to a reduction in α-galactosidase staining.

In summary, the yeast two-hybrid results support the notion that CMM2 monomer association *in vivo* mostly depends on the stability of the coiled-coil. The reduced interaction observed with the CMM2-mut2 fragment indicates that the N-terminus of CMM2 may also contribute to the self-interaction, albeit to a lower degree. In addition, the slight reduction in α-galactosidase staining observed with the CMM2-mut3 construct suggests that the mutated residues, which were possibly involved in CMM2 multimerization through crystal lattice formation, are only partially responsible for the stability of the coiled-coil structure.

### Multimerization of CMM2 is required for its silencing activity

**Figure 4:**
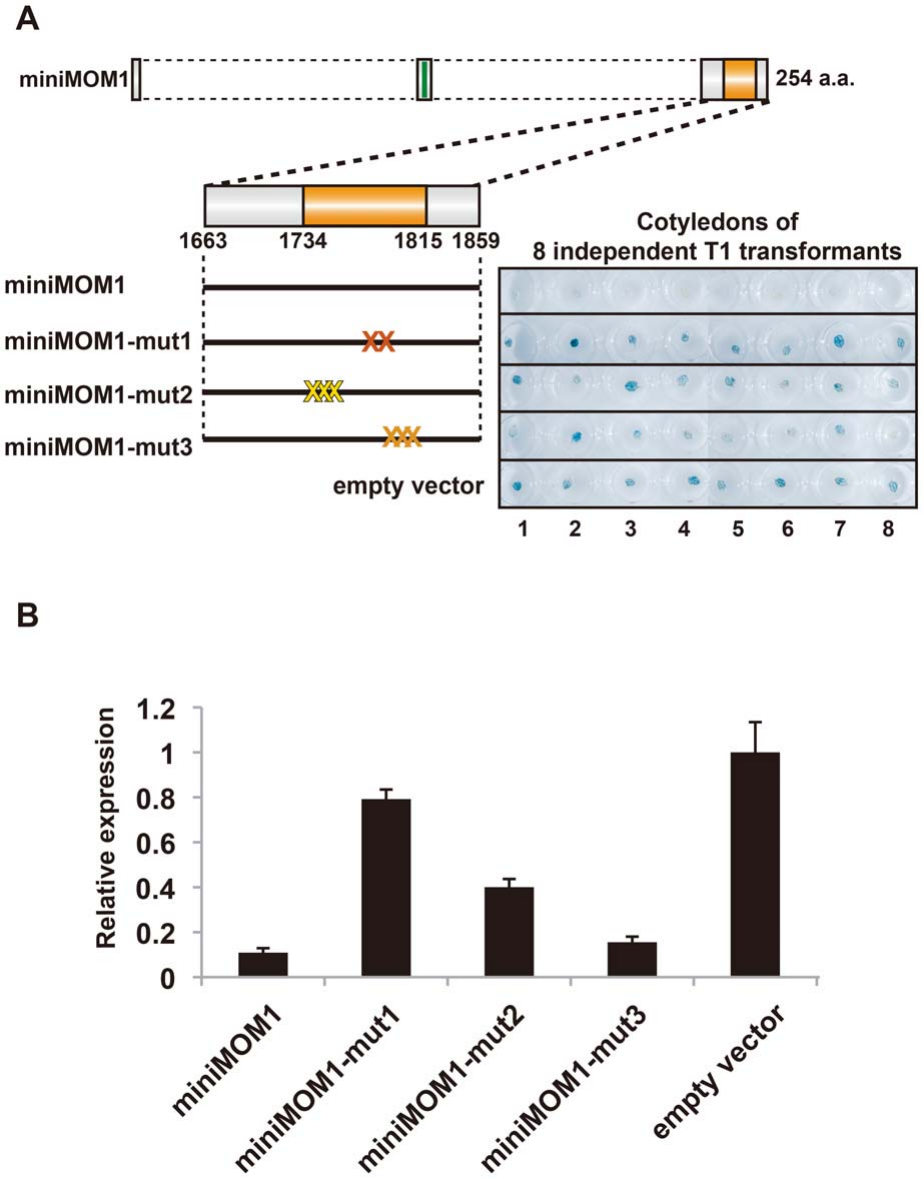
Multimerization of CMM2 domains are crucial for TGS activity. (A) *Left*, schematic models of transgene constructs used for transgenic complementation assays in *mom1* L5 plants. “X” represents mutations colored as in Figure 3. *Right*, histochemical GUS staining of cotyledons of eight independent 1-week-old T1 transgenic plants transformed with the corresponding mutant derivatives of miniMOM1. The “empty vector” control corresponds to *mom1* L5 transformed with a vector construct without miniMOM1. (B) Relative levels of *GUS* mRNA in T2 plants from 3 independent T1 plants determined by quantitative RT-PCR and normalized to 18S rRNA. The mean of the “empty vector” control was set to 1. Error bars represent S.E. calculated from 3 experimental sets of 40 to 50 plants each.

The results of structural analyses of CMM2 interactions supported by yeast two-hybrid results prompted us to determine whether multimerization of CMM2 is also essential for TGS activity. To address this question, we transformed *mom1*L5 plants with miniMOM1 constructs harboring the same point mutations examined for their interaction in yeast (Figure 4A). For each construct, multiple transgenic *mom1* L5 plants were generated and subjected to histochemical staining for GUS activity (eight T1 plants for each construct) (Figure 4A). Subsequently, T2 progeny of three randomly chosen T1 plants were examined by quantitative RT-PCR to determine levels of *GUS* mRNA (Figure 4B).

All three mutations caused a clear reduction in miniMOM1 silencing activity (Figure 4A and 4B). Importantly, the degree of TGS release correlated well with results obtained in the yeast two-hybrid assays. In both experiments, CMM2-mut1 had the strongest influence, practically abolishing the TGS activity of CMM2 (Figure 4A and 4B). In contrast, CMM2-mut2 retained partial TGS activity, although significantly lower than that of the intact CMM2 fragment (Figure 4A and 4B). In both cases, the decrease in TGS activity was not due to reduced protein stability (Figure S2). Finally, the CMM2-mut3 had TGS activity comparable to the original CMM2, as revealed by quantitative RT-PCR (Figure 4B), but displayed increased variability in the degree of TGS among individuals examined by histochemical GUS assays (Figure 4A). Obviously, this variability may reflect differences between the eight independent transgenic events, perhaps further exaggerated by a GUS protein half-life longer than that of *GUS* mRNA. Nevertheless, all the above results are consistent with the notion that CMM2 homodimerization, through the formation of a stable coiled-coil structure, is a prerequisite for TGS activity.

### The target-specific contribution of CMM2 to MOM1-mediated TGS

Having demonstrated that a short CMM2 domain and its intermolecular interactions are essential for TGS at the *GUS* transgenic locus, we next extended our analysis to endogenous genes normally regulated by MOM1. MOM1 targets have been assigned to three different classes based on the degree of MOM1 cooperation with RdDM [22], [23]. At the *SDC* locus (*At2g17690*), TGS requires both MOM1 and RdDM acting epistatically [22], [23]. At the *APUM9* locus (*At1g35730*) and at various transgenic loci, TGS requires MOM1 and RdDM acting independently [23]. Finally, at the *MULE-F19G14* (*At2g15810*), *At3g42719* and *At2g11780* loci, TGS control requires almost exclusively MOM1, although marginal contribution of RdDM cannot be ruled out [22], [23].

**Figure 5:**
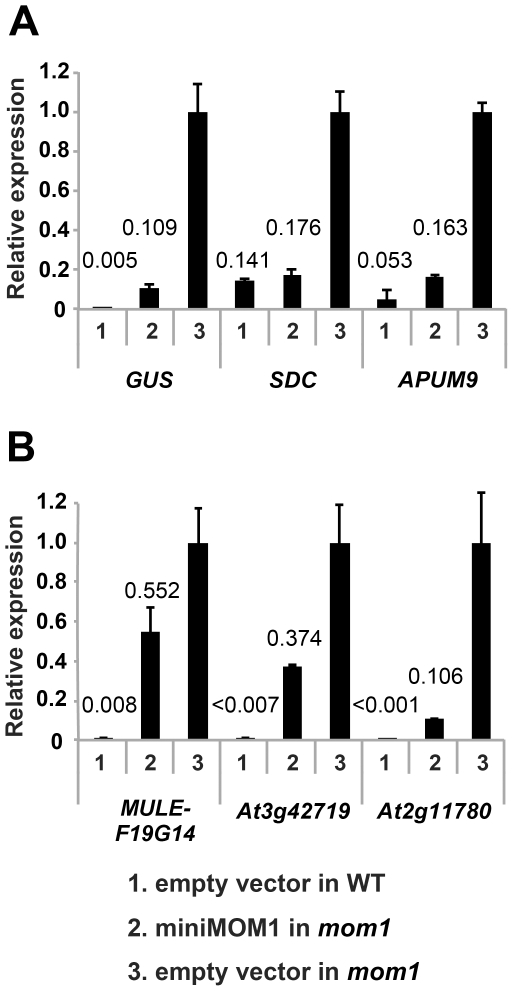
CMM2 acts selectively on MOM1-regulated TGS targets. Relative levels of mRNAs in T2 plants of various MOM1 target loci determined by quantitative RT-PCR and normalized to 18S rRNA. The targets regulated by MOM1 co-operatively with RdDM (A) and largely by MOM1 only (B) were investigated. These T2 plants were delivered from 3 independent T1 plants. The mean of “empty vector in *mom1*” was set to 1. The mean values of relative expression are indicated above columns. Error bars represent S.E. calculated from 3 experimental sets of 40 to 50 plants each.

In transgenic complementation assays, we determined the transcripts levels for each of the loci described above in wild-type *Arabidopsis*, in the *mom1* mutant and in *mom1* strains complemented by miniMOM1 (Figure 5, “empty vector in WT”, “empty vector in *mom1”* and “miniMOM1 in *mom1”*, respectively) and also calculated miniMOM1 silencing efficiency for each chromosomal target (Figure S3). miniMOM1 was clearly more effective in silencing *SDC*, *APUM9* and the transgenic *GUS* locus (targets requiring MOM1 and RdDM for TGS) than for silencing of *MULE-F19G14*, *At3g42719* and *At2g11780* (mostly dependent on MOM1 activity for their TGS). Interestingly, although the above trend was apparent, we observed variations in silencing efficiency within each category of the loci. For example transgenic *GUS* locus was silenced less efficiently than *SDC* or *APUM9* (Figure 5A). It has been observed that *APUM9* and *SDC* have clearer RdDM dependence for their silencing [22], [23], [30] than this documented for the *GUS* locus [31]. We noticed also certain variation in miniMOM1 silencing efficiency in the second category of loci (*MULE-F19G14, At3g42719* and *At2g11780*), which could also reflect marginal but variable contribution of RdDM to their TGS (Figure 5B). Nevertheless, despite of the observed and somehow expected variations in the functional overlaps between MOM1 and RdDM mediated TGS pathways, our results suggest that miniMOM1, and therefore the CMM2 domain, is mostly sufficient to replace MOM1 function at chromosomal targets that are co-regulated by MOM1 and RdDM, but does this much less efficient at targets regulated by MOM1, mostly independently of RdDM.

**Figure 6:**
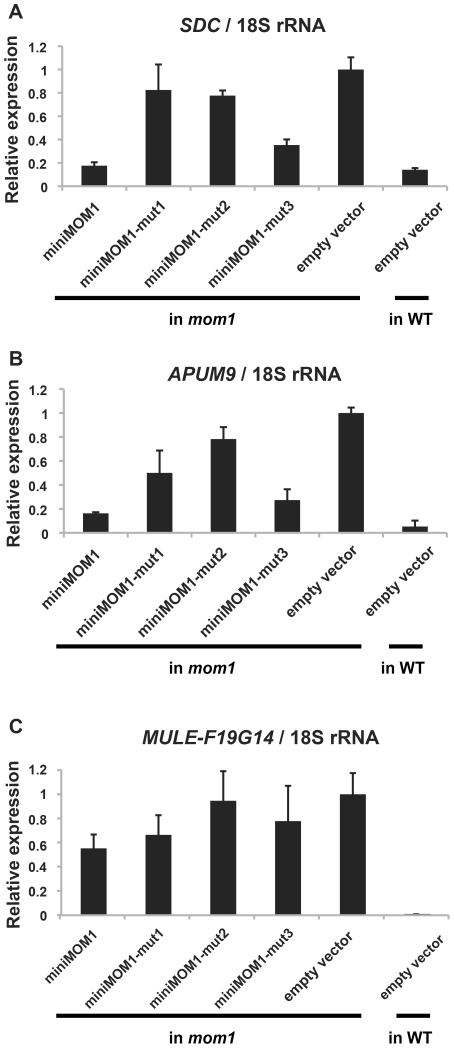
Multimerization of CMM2 domains is crucial for TGS activity at various chromosomal loci. Relative levels of mRNAs in T2 plants of various MOM1 target loci determined by quantitative RT-PCR and normalized to 18S rRNA. These T2 plants were delivered from 3 independent T1 plants. The mean of “empty vector in *mom1*” was set to 1. Error bars represent S.E. calculated from 3 experimental sets of 40 to 50 plants each.

The necessity and sufficiency of CMM2 for miniMOM1 activity at these various chromosomal targets was further assessed by transgenic complementation with miniMOM1 deletion constructs. CMM2 was seen to be essential for TGS regulation at two loci co-regulated by RdDM (Figure S4). Moreover, we examined whether TGS at these loci requires CMM2 multimerization (Figure 6). Consistent with the TGS regulation observed at the transgenic *GUS* locus (Figure 4), miniMOM1-mut1 and miniMOM1-mut2 displayed a significant reduction in silencing activity also at *SDC* and *APUM9*. However, at *MULE-F19G14*, the CMM2 mutations had only a marginal effect due to the generally limited role of CMM2 in silencing this locus (Figure 6).

## Discussion

MOM1, as a large nuclear protein involved in chromatin-mediated transcriptional regulation, is able to shift transcriptional states largely independently of changes in epigenetic marks [17], [19], [20]. It was shown previously that a short fragment of MOM1 of 198 amino acids (miniMOM1) containing a CMM2 domain of 82 amino acids (1734 to 1815) is necessary and sufficient to control transcriptional suppression at a transgenic locus and at endogenous pericentromeric sequences derived from *Athila* retroelements [24]. These results indicated that CMM2, which is conserved in all vascular plants, is essential for MOM1-mediated TGS. To gain further insight into molecular mechanisms associated with CMM2 activity in silencing, we performed structural and functional analyses. An important prerequisite for such studies was an assessment of CMM2 silencing activity in the absence of its flanking sequences. Interestingly, deletions of either the N or C terminal flank had no influence on the TGS activity of miniMOM1; however, after simultaneous deletion of both flanks, the silencing activity of the CMM2 construct was reduced by approximately 20% due to variable transcript levels in transgenic plants seen for this particular construct. Hence, the majority of miniMOM1 silencing activity can be clearly attributed to CMM2. However, each of its flanks can act to support CMM2-mediated TGS by stabilization of transgenic expression or possibly further stabilizing CMM2 multimerization discussed below. Since the central role of CMM2 in miniMOM1-mediated regulation of TGS is apparent, we decided to elucidate its structural and functional features.

The CMM2 structure presented here constitutes the first example of an anti-parallel coiled-coil containing multiple hendecad repeats. Although three structures of hendecad-based coiled-coil are reported in the literature (cytoskeleton protein [29]; tetrabrachion [32]; H+/ATPASE [33]), available knowledge is rather limited about the oligomeric properties of hendecad-containing coiled-coil structures as compared to heptad-based coiled-coils. The CMM2 structure thus provides additional and important information on this particular type of coiled-coil. Hendecad-containing structures form dimers or tetramers. The CMM2 fragment forms dimeric coiled-coils with extensive hydrogen bonds or salt bridges mediating inter-chain contacts via residues at positions g and k. This is in marked contrast to the recently reported parallel hendecad-based coiled-coil structure of the peripheral stalk of the H^+^-ATPase/synthase, where inter-chain contacts are mostly due to residues at positions d, e and i [33].

Importantly, the formation of a coiled-coil by CMM2 domains seems to occur *in vivo*, where it is essential for TGS activity. Since residues at positions e and i of CMM2 are not involved in inter-chain contacts, they may mediate interactions with MOM1 partners *in vivo*. This raises the possibility that the CMM2 coiled-coil structure is a “landing platform” for additional factors, although such additional interaction partners have not yet been described. This resembles the recently reported homodimerization of the coiled-coil domain of barley MLA protein, which belongs to the family of R proteins involved in cellular responses to pathogen infection [34]. It has been shown that homodimerization of only the coiled-coil domains of MLA can activate programmed cell death, which is essential for the defense response to pathogens. This homodimerization of MLA coiled-coil domains provides a binding platform for WRKY transcription factors and it was proposed that homodimers of MLA coiled-coil domains constitute the minimal R protein unit able to initiate the cell death response [34]. Similarly, dimerization of the CMM2 of MOM1 is necessary and sufficient for its TGS activity and mutations altering this process abolish TGS mediated by CMM2. It is also possible that the multimerization of the CMM2 domain, either via the coiled-coil structure or via the N- and C-termini could contribute to its interaction with other proteins. The functional data obtained for CMM2 structure-guided mutants will make it possible to design an optimized strategy for revealing further proteins with a binding affinity specific for the CMM2 coiled-coil domain and subsequently to assess their contributions to MOM1-mediated TGS. Moreover, the charged amino acids located at positions e and i may also be involved in nucleic acid recognition. Further experiments are needed to determine the veracity of these clues.

In a forward genetic screen for modifiers of the silencing properties of the *mom1* mutant, a mutation in the RdDM component, which is a plant-specific RNA polymerase V (*nrpe1*), was isolated as an enhancer of TGS release observed in *mom1* [23]. This provided evidence for the functional interaction of silencing mechanisms mediated by MOM1 and RdDM pathways. Moreover, the results from previous studies [24] and those presented here show that miniMOM1-mediated TGS at chromosomal targets co-regulated by MOM1 and RdDM (transgenic locus, *SDC*, *APUMP9*) is very effective. These observations substantiated the evidence for a critical role of the CMM2 domain in this process. Surprisingly, however, with the chromosomal targets *MULE-F19G14*, *At3g42719*, and *At2g11780* for which TGS seems to be regulated by MOM1 without significant RdDM assistance, miniMOM1 was not able to initiate and/or maintain TGS. These results imply that, in addition to CMM2, further domains of MOM1 are essential for the establishment and the maintenance of TGS at *MULE-F19G14*, *At3g42719*, *At2g11780* and possibly other loci that are controlled by MOM1 in a fashion largely independent of RdDM. The slight reduction in *MULE-F19G14* transcript levels observed upon introduction of miniMOM1 can be explained by a minor contribution of RdDM to *MULE-F19G14* silencing, as revealed by slight release of its silencing in the *nrpd1* mutant (mutated in plant specific Pol IV) [22], in which the biogenesis of siRNAs required for RdDM is impaired. Similarly, the reduction in the *At3g42719* and *At2g11780* transcript levels may be explained by a minor contribution of RdDM to their TGS.

As a general conclusion, we propose that CMM2, although it seems to be necessary for MOM1-mediated TGS, is only sufficient for silencing at loci that are evidently co-regulated by MOM1 and RdDM. In other words, the cooperation of MOM1 with RdDM, which was initially revealed by their genetic interaction [23], seems to be largely mediated by CMM2 homodimerization. While MOM1 orthologs containing CMM2 are present in vascular plants, the RdDM-related pathway is more extensive and has also been documented in mosses [35]. This suggests that the two TGS mechanisms evolved independently and that MOM1, with its CMM2-mediated silencing activity, augmented the RdDM pathway at the onset of the vascular plants lineage. This hypothesis is further supported by the observation that TGS at certain loci remains under RdDM control in a MOM1-independent fashion [23]. The question of why only some RdDM targets require MOM1, and especially the CMM2 domain, to support RdDM-mediated TGS remains open.

As presented here, the results suggest that MOM1 can use distinct and target-specific TGS mechanisms that can be assigned to its structural features. This is consistent with previous genetic studies that revealed the target-dependent multi-functional nature of MOM1 [22], [23]. Importantly, here we are able to relate MOM1 silencing activity to a short but essential CMM2 domain and to its ability to form a hendecad-based coiled-coil involved in intermolecular interactions. This very short CMM2 protein fragment seems to be a dimeric platform especially critical for the cooperation of MOM1 with the RdDM pathway.

## Materials and Methods

### Plant material

All plants used were in the Columbia accession. L5 *GUS* line and *mom1-3* (SALK_141293) were described before [25], . Transgenic strains were obtained by *Agrobacterium* containing a modified pGWB-NB1 binary vector which is a prototype of pGWB601 [37] supplemented with a *BAR* selectable gene. The fragment containing approximately 2 kb of *MOM1* promoter linked to a coding sequence of miniMOM1 [24] was subcloned into pDONR-zeo by Gateway BP reaction (Life Technologies). miniMOM1 deletion and mutant derivatives were obtained by inverse PCR using a KOD hotstart DNA polymerase (Novagen), and subcloned by LR reaction (Life Technologies) into a pGWB-NB1 binary vector. The constructs were transformed into *mom1-3* L5 plants using floral dip methods [38].

### Histochemical GUS analyses

Cotyledons of 7-day-old seedlings (one cotyledon per plant) were vacuum infiltrated for 30 min with X-Gluc solution [400 µg/ml 5-bromo-4-chloro-3-indolyl-β-glucuronide, 3 mM K_3_Fe(CN)_6_, 3 mM K_4_Fe(CN)_6_, 10 mM ethylenediamine tetraacetic acid and 100 mM Na-phosphate buffer, pH 7.0] and incubated 2 overnights at 37°C. The chlorophyll was removed by extraction in 70% ethanol.

### Quantitative RT–PCR and Western blot analyses

Total cellular RNA was isolated from 7-day-old seedlings using the TRI reagent (Sigma) according to the manufacturer's instructions. After RQ1 DNase treatment (Promega), the first-strand cDNA was synthesized with Superscript VILO cDNA synthesize kit (Life Technologies). Real time PCR reactions were performed with fluorescent probes using QuantiFast Multiplex PCR kit (Qiagen) in ABI7900FT (Life technologies). *GUS* and *APUM9* transcripts were subjected to duplex analyses using two different fluorescent dyes, while *SDC*, *MULE-F19G14*, *At3g42719* and *At2g11780* RNAs were detected by separate reactions with simplex analyses for each dye. Amounts of mRNA were calculated by subtraction of the values obtained without reverse transcriptase reaction and normalized with respect to the amount of 18S rRNA. Primers and probes used for RT-PCR were designed with Primer Express program (Life technology) and their sequences are listed in Table S2.

### Yeast two-hybrid assay

Yeast two-hybrid assay was performed by Matchmaker Gold Yeast Two-Hybrid System (Clontech) according to the manufacturer's instruction.

### Western blotting

Western blotting was performed as described in [24].

### Expression, purification, and crystallization

A construct encoding a part of the MOM1 protein (aa 1700 to 1814) was overexpressed in *E. coli* and the protein was purified as previously reported [26], concentrated to ∼15 to 18 mg/ml and crystallized at 4°C via hanging drop vapor diffusion, with initial crystals forming in 0.1 M Tris pH 8.5, 0.3 M magnesium formate dihydrate buffer. Crystallization was further optimized and, after stabilization in similar conditions, was supplemented by 20% ethylene glycol. The crystals were flash frozen in liquid nitrogen and their diffraction properties measured previously described [26]. A seleno-methionine-containing protein was produced using *E. coli* strain 834, which is auxotrophic for methionine, in minimal medium supplemented with seleno-methionine. Crystals of proteins with seleno-methionine grew in conditions similar to those for the native protein and were subjected to a similar stabilization procedure before freezing.

### Data collection and processing

Diffraction data were collected at the European Synchrotron Radiation Facility (ESRF, Grenoble, France) on the beam lines ID23-1, ID29 and ID14-4. A complete data set using native protein crystal was collected at a maximum resolution of 3.2 Å. A further data set was collected at the selenium edge with a selenium-containing protein crystal. This crystal diffracted to ∼3.5 Å resolution. A Single Anomalous Dispersion phasing procedure was used to solve the phase problem using the selenium as heavy atoms. Briefly, the 8 seleno-methionines were located by the program SHELXD using the measured anomalous signals [39]–[41]. The sites were subsequently injected into the experimental SAD phasing procedure as defined in SHARP [42]. Density modification was then used to improve the initial set of phases [43]. Long tubes of electron density were readily visible in the calculated electron density map and the initial model was built using the program Coot [44] with an alanine-only model. The protein register was defined based on the position of the methionine residues. Several rounds of refinement/rebuilding were done iteratively using the PHENIX software [45]. All the built residues are in the favored and allowed regions of the Ramachandran plot. Residues 1700 to 1728 and 1811 to 1814 are disordered or very poorly ordered in every CMM2 molecules. The protein coordinates have been deposited in the Protein Databank with the PDB code 3VEM.

### Accession numbers

AtMOM1 (AAF73381); PtMOM1 (EEE94860); VvMOM1 (CBI16337); OsMOM1 (EEE64938); PtaMOM1 (Co364249); ZmMOM1 (GRMZM2G47428); AcMOM1 (AcoGoldSmith_v1.001036m); MtMOM1 (fpc265_22); SmMOM1 (EFJ29853).

## Supporting Information

Figure S1 (A) Relative levels of *GUS* mRNA. Levels of *GUS* mRNA determined by quantitative RT-PCR and normalized to 18S rRNA. For each construct 40-50 progeny plants of independent T1 transgenics (numbers 1, 2, and 3, which are corresponding with those of Figure 1B) transformed with deletion derivatives of miniMOM1 were used for RNA isolation. Error bars represent S.E. calculated from 2 technical replicates. The mean of technical replicates of T1 plant no. 1 transformed with “empty vector” was set to 1. (B) Transcript levels of various *miniMOM1* derivatives. *Top*, semi-quantitative RT-PCR revealing the levels of *miniMOM1* transcripts and its deletion derivatives in the same RNA used in (A). *Bottom*, *ACT2* transcripts as internal controls. RT+ and RT−, reactions with presence or absence of reverse-transcriptase, respectively.
(TIF)

Figure S2 Mutant derivatives of miniMOM1 protein are stably expressed. (A) Relative levels of *GUS* mRNA determined by quantitative RT-PCR and normalized to 18S rRNA. For each construct 40–50 progeny plants of independent T1 transgenics (A and B) transformed with mutant derivatives of miniMOM1 were used for RNA isolation. Error bars represent S.E. calculated from 2 technical replicates. The mean of technical replicates of T1 plant B transformed with “empty vector” was set to 1. (B) *Top*, western blot revealing the levels of HA-tagged miniMOM1 and its mutant derivatives in 1-week-old T2 plants whose siblings were used in (A). *Bottom*, Coomassie Brilliant Blue-stained parallel gel.
(TIF)

Figure S3 Relative silencing efficiency (SE) of miniMOM1-mediated TGS at various chromosomal targets. (A) Silencing efficiency at MOM1 targets regulated in cooperation with RdDM and (B) silencing efficiency at MOM1 targets regulated mostly by MOM1 alone. Columns marked (1) “SE in WT “ (set to 100%), and columns marked (2) “SE in *mom1* complemented by miniMOM1*”* were calculated as ratios of the relative expression in “empty vector in WT” and in “miniMOM1 in *mom1*” (Figure 5). The values of “SE in *mom1* complemented by miniMOM1” are shown above columns.
(TIF)

Figure S4 TGS activity of the CMM2 domain for chromosomal targets. Relative levels of mRNAs in T2 plants of various MOM1 target loci determined by quantitative RT-PCR and normalized to 18S rRNA. These T2 plants were delivered from 3 independent T1 plants. The mean of “empty vector in *mom1*” was set to 1. Error bars represent S.E. calculated from 3 experimental sets of 40 to 50 plants each.
(TIF)

Table S1 Crystallographic data collection and refinement statistics.
(DOC)

Table S2 List for primers and probes used for RT–PCR.
(DOC)
