# Supplementary figures and images for: Structural Basis of Transcriptional Gene Silencing Mediated by Arabidopsis MOM1
Source: PLoS Genet. 2012 Feb 9;8(2):e1002484. doi: 10.1371/journal.pgen.1002484 (PMC3276543; doi:10.1371/journal.pgen.1002484)

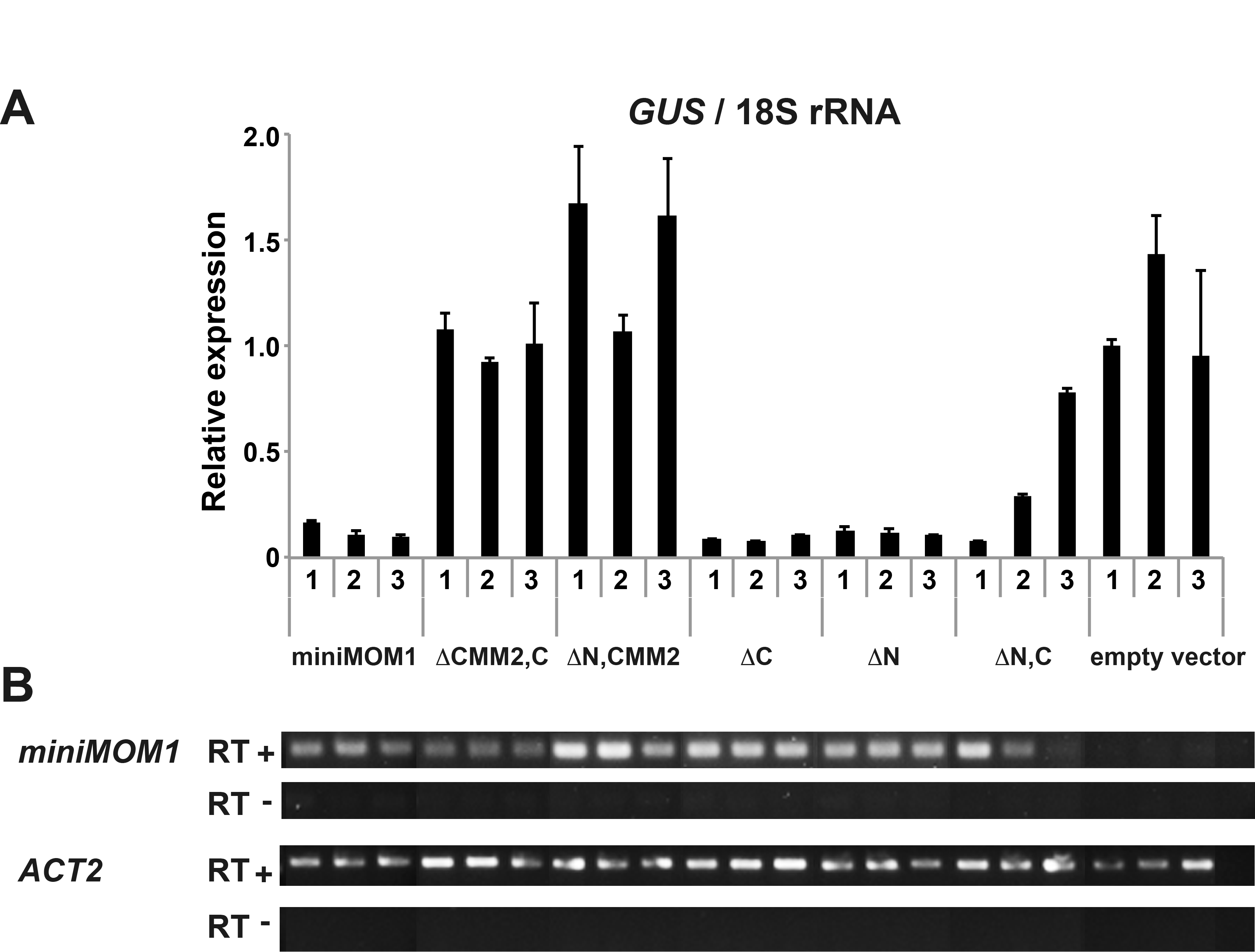

Supplement: Figure S1 — (A) Relative levels of GUS mRNA. Levels of GUS mRNA determined by quantitative RT-PCR and normalized to 18S rRNA. For each construct 40-50 progeny plants of independent T1 transgenics (numbers 1, 2, and 3, which are corresponding with those of Figure 1B) transformed with deletion derivatives of miniMOM1 were used for RNA isolation. Error bars represent S.E. calculated from 2 technical replicates. The mean of technical replicates of T1 plant no. 1 transformed with “empty vector” was set to 1. (B) Transcript levels of various miniMOM1 derivatives. Top, semi-quantitative RT-PCR revealing the levels of miniMOM1 transcripts and its deletion derivatives in the same RNA used in (A). Bottom, ACT2 transcripts as internal controls. RT+ and RT−, reactions with presence or absence of reverse-transcriptase, respectively. (TIF) [file pgen.1002484.s001.tif]

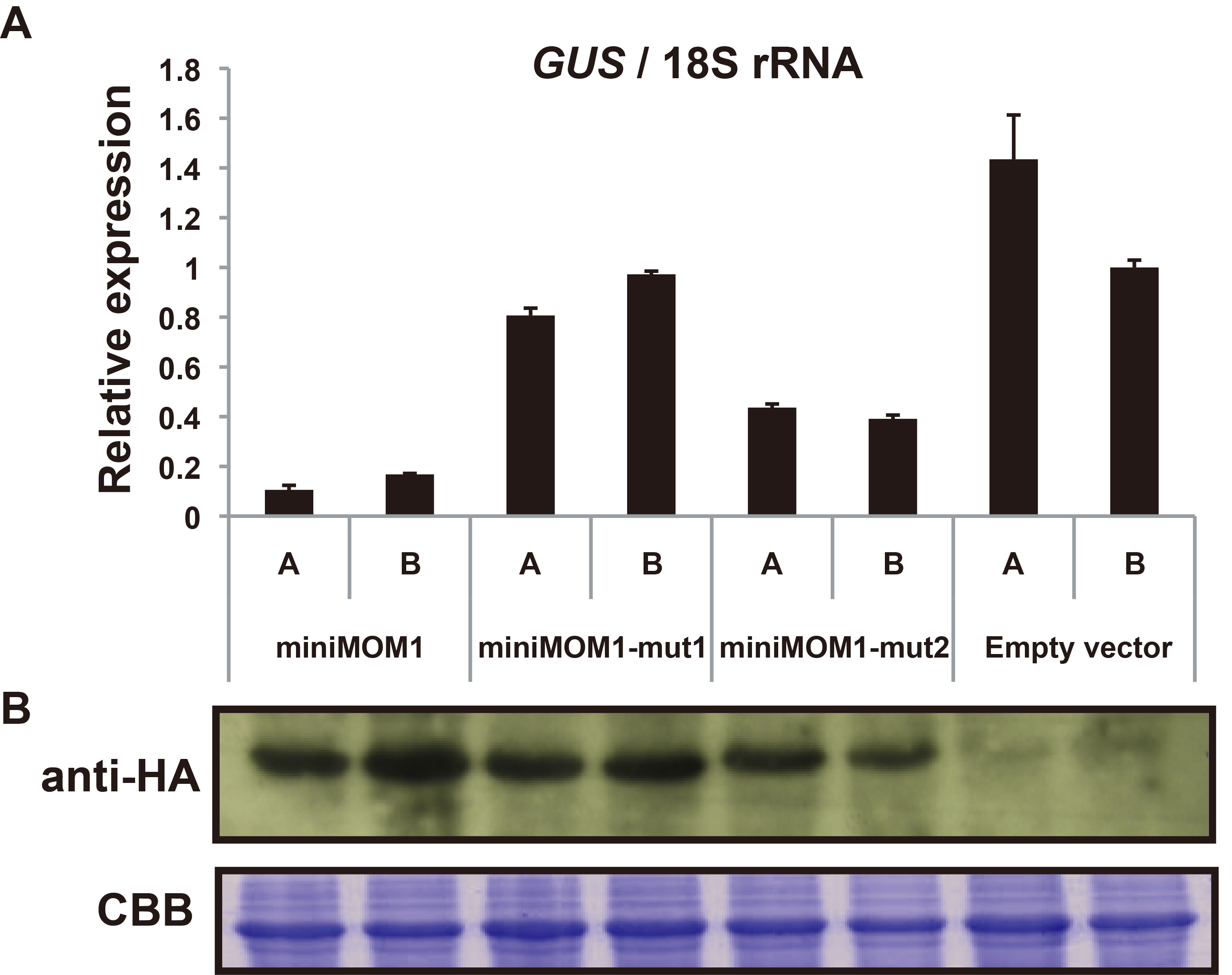

Supplement: Figure S2 — Mutant derivatives of miniMOM1 protein are stably expressed. (A) Relative levels of GUS mRNA determined by quantitative RT-PCR and normalized to 18S rRNA. For each construct 40–50 progeny plants of independent T1 transgenics (A and B) transformed with mutant derivatives of miniMOM1 were used for RNA isolation. Error bars represent S.E. calculated from 2 technical replicates. The mean of technical replicates of T1 plant B transformed with “empty vector” was set to 1. (B) Top, western blot revealing the levels of HA-tagged miniMOM1 and its mutant derivatives in 1-week-old T2 plants whose siblings were used in (A). Bottom, Coomassie Brilliant Blue-stained parallel gel. (TIF) [file pgen.1002484.s002.tif]

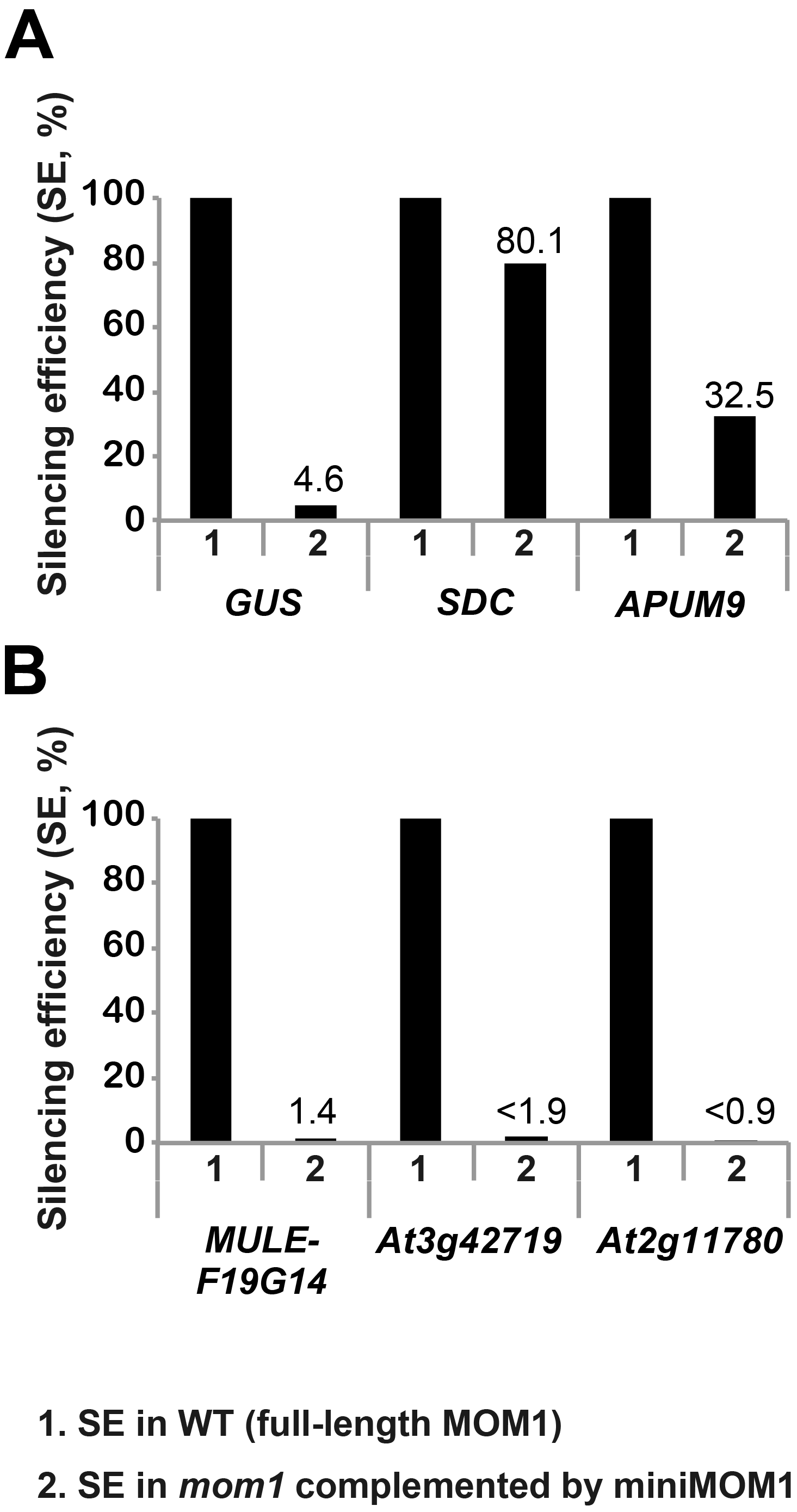

Supplement: Figure S3 — Relative silencing efficiency (SE) of miniMOM1-mediated TGS at various chromosomal targets. (A) Silencing efficiency at MOM1 targets regulated in cooperation with RdDM and (B) silencing efficiency at MOM1 targets regulated mostly by MOM1 alone. Columns marked (1) “SE in WT “ (set to 100%), and columns marked (2) “SE in mom1 complemented by miniMOM1” were calculated as ratios of the relative expression in “empty vector in WT” and in “miniMOM1 in mom1” (Figure 5). The values of “SE in mom1 complemented by miniMOM1” are shown above columns. (TIF) [file pgen.1002484.s003.tif]

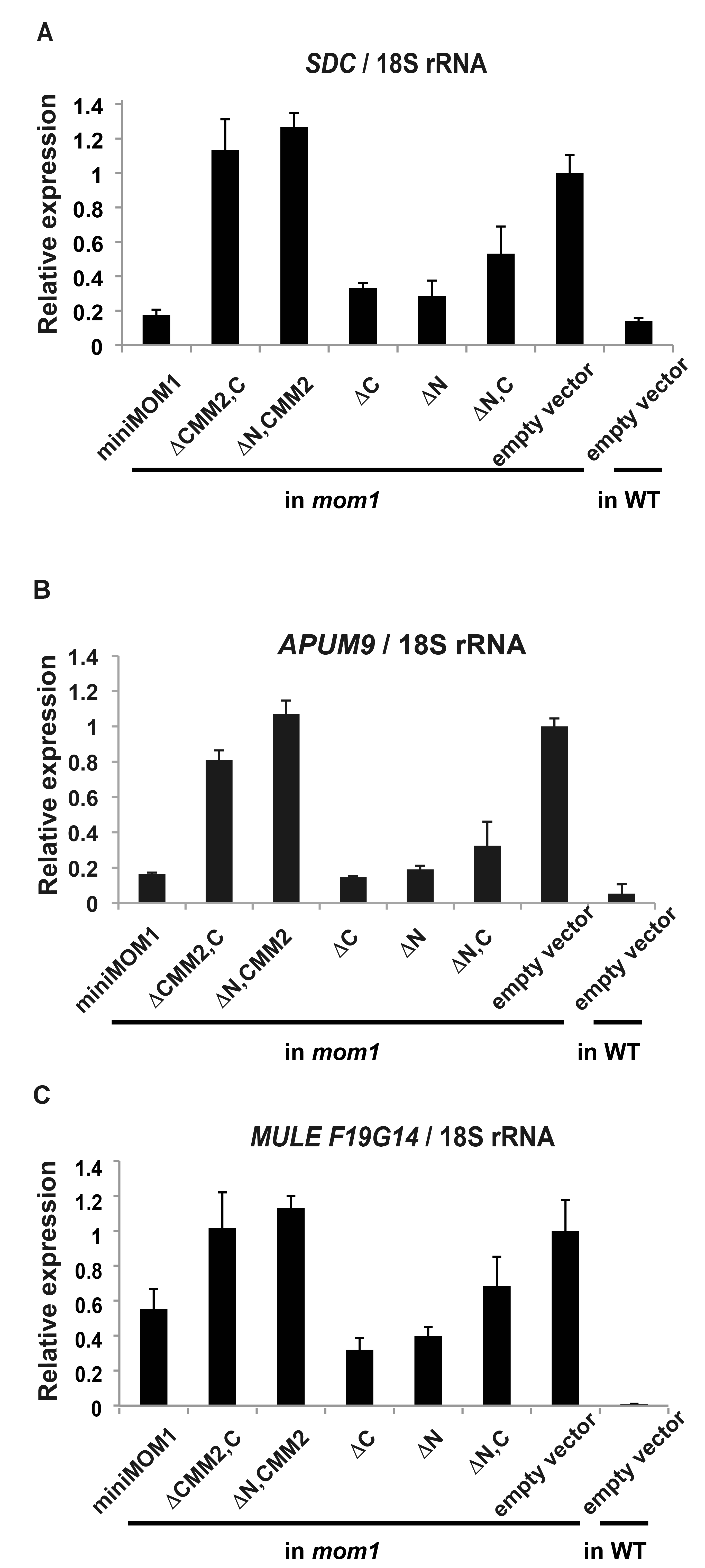

Supplement: Figure S4 — TGS activity of the CMM2 domain for chromosomal targets. Relative levels of mRNAs in T2 plants of various MOM1 target loci determined by quantitative RT-PCR and normalized to 18S rRNA. These T2 plants were delivered from 3 independent T1 plants. The mean of “empty vector in mom1” was set to 1. Error bars represent S.E. calculated from 3 experimental sets of 40 to 50 plants each. (TIF) [file pgen.1002484.s004.tif]
