# Supplementary material for: Structural Basis of Transcriptional Gene Silencing Mediated by Arabidopsis MOM1
Source: PLoS Genet. 2012 Feb 9;8(2):e1002484. doi: 10.1371/journal.pgen.1002484 (PMC3276543; doi:10.1371/journal.pgen.1002484)
Supplement: Table S1 — Crystallographic data collection and refinement statistics. (DOC) [file pgen.1002484.s005.doc]

**Table S1 Crystallographic data collection and refinement statistics.**

| *Data collection* |  |
| --- | --- |
| Space group | *P*3_1_21 |
| Cell dimensions (Å) | a = b = 85.64,c = 292.74 |
| Resolution range (Å) | 50.0 - 3.2 |
| R_merge_(F) (%) | 0.072 (0.893) |
| *I/σ(I)* | 20.56 (2.15) |
| Completeness (%) | 99.5 (96.6) |
| Redundancy | 21.7 |
|  |  |
| *Refinement* |  |
| Reflections | 21107 |
| *R_work_/R_free_* | 25.8 / 29.3 |
| RMSD |  |
| Bond length | 0.01 |
| Bond angle | 1.26 |
| No. of protein atoms | 2761 |
| Overall B factor | 112 |

*R*_mrgd-F_ is the quality of the reduced structure factor amplitudes according to [46].

*R*_mrgd-F_ =
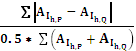
 with A_I_ =
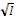
 if I ≥ 0 or A*_I_* = -
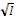
 if I < 0. I_h,P_ =
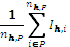
 and I_h,Q_ =
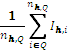
.
