# Supplementary material for: Structural Basis of Transcriptional Gene Silencing Mediated by Arabidopsis MOM1
Source: PLoS Genet. 2012 Feb 9;8(2):e1002484. doi: 10.1371/journal.pgen.1002484 (PMC3276543; doi:10.1371/journal.pgen.1002484)
Supplement: Table S2 — List for primers and probes used for RT–PCR. (DOC) [file pgen.1002484.s006.doc]

**Table S2 List for primers and probes used for RT-PCR.**

| Name | Sequence (5'-3') | Fluorescent dye |
| --- | --- | --- |
| 18Sr_TM_probe | CCGCCCGTCGCTCCTACCGAT | JOE |
| 18Sr_TM_FW | CGTCCCTGCCCTTTGTACAC |  |
| 18Sr_TM_RV | CGAACACTTCACCGGATCATT |  |
| GUS_TM_probe | CCTTTGCCACGCAAGTCCGCA | FAM |
| GUS_TM_FW | CACCATCAGCACGTTATCGAA |  |
| GUS_TM_RV | CTGGCTTTGGTCGTCATGAA |  |
| SDC_TM_probe | TCGCGCACAAGCAAACGGACA | FAM |
| SDC_TM_FW | AAGCCGCTCCAAATCATTCTC |  |
| SDC_TM_RV | CGTGCCATGATACTTGCTCAA |  |
| APUM9_TM_probe | CCCGTACCTGTAAACCTTCGCCACCA | FAM |
| APUM9_TM_FW | TGCAGTAAGGGCTCGAGTGTAG |  |
| APUM9_TM_RV | CCAAAGGACCTGTTCGTGCTA |  |
| MULE-F19G14_TM_probe | CCTCCCTAGGCGATGCTTCCTCGA | FAM |
| MULE-F19G14_TM_FW | TCCGGCGAGTTCGATTTC |  |
| MULE-F19G14_TM_RV | GGTGTGATCCTTCTAGCCTTTGTG |  |
| At3g42719_TM_probe | CCATTCCGCACTCAACCAGACACTTG | FAM |
| At3g42719_TM_FW | CGACTCCCAGCTTCAGAGTCTT |  |
| At3g42719_TM_RV | CTGGAGTCTTCTTCTCTTCCTTGTG |  |
| At2g11780_TM_probe | AAGAGACCCAAACCACCGACTTATGG | FAM |
| At2g11780_TM_FW | TTTGCAACACATCTTCCACATG |  |
| At2g11780_TM_RV | CGAAGAAGGAGCATCCACATC |  |
| miniMOM1_RT_FW | CCTAGCAGCTTCTTCGAAACG |  |
| miniMOM1_RT_RV | TCCACCATCGCCAGTTTTG |  |
